# Argovit™ Silver Nanoparticles Mitigate Sodium Arsenite-Induced Cytogenotoxicity Effects in Cultured Human Lymphocytes

**DOI:** 10.3390/toxics13070539

**Published:** 2025-06-27

**Authors:** María del Carmen Jauregui Romo, Balam Ruiz Ruiz, Francisco Casilas-Figueroa, Nayeli Guadalupe Girón Vázquez, Roberto Luna Vázquez Gómez, Olivia Torres-Bugarín, Idalia Yazmín Castañeda Yslas, Alexey Pestryakov, Nina Bogdanchikova, María Evarista Arellano García

**Affiliations:** 1 Facultad de Ciencias, Universidad Autónoma de Baja California, Ensenada 22860, Mexico; cjauregui@gmail.com (M.d.C.J.R.); nayeli.giron@uabc.edu.mx (N.G.G.V.); 2 Facultad de Ciencias de la Salud, Universidad Autónoma de Baja California, Ensenada 22890, Mexico; casillas.francisco@uabc.edu.mx (F.C.-F.); rluna@uabc.edu.mx (R.L.V.G.); 3 Departamento de Medicina Interna II, Decanato de Facultad de Medicina, Universidad Autónoma de Guadalajara, Zapopan 45129, Mexico; oliviatorres@hotmail.com; 4 Centro de Nanociencias y Nanotecnología, Universidad Nacional Autónoma de México, Ensenada 22860, Mexico; icastaneda@ens.cnyn.unam.mx (I.Y.C.Y.); nina@ens.cnyn.unam.mx (N.B.); 5 Research School of Chemistry and Applied Biomedical Sciences, Tomsk Polytechnic University, 634050 Tomsk, Russia; pestryakov2005@yandex.ru

**Keywords:** cytotogenotoxicity, lymphocytes culture, silver nanoparticles

## Abstract

Exposure to arsenic, a known environmental and occupational genotoxicant, poses significant health risks. Identifying agents capable of mitigating its effects is crucial for public health. This study evaluates the protective potential of Argovit™ silver nanoparticles (AgNPs) against cytotoxic and genotoxic damage induced by sodium arsenite in ex vivo cultured human lymphocytes obtained from the whole blood of healthy donors. Lymphocytes were exposed to sodium arsenite (3.7 × 10^−3^ µg/mL) and Argovit™ AgNPs (3.6 × 10^−3^ µg/mL). The cytokinesis-block micronucleus (CBMN) assay was performed using a modified 144 h protocol to assess delayed effects across two cell cycles. Four groups were analyzed: untreated control, sodium arsenite only, AgNPs only, and sodium arsenite followed by AgNPs. Arsenite exposure increased cytotoxic and genotoxic biomarkers. In contrast, post-treatment with AgNPs significantly reduced these effects. All treatments were performed in duplicate, and data were analyzed using the Kruskal–Wallis test with Dunn’s post hoc comparison (*p* < 0.05). Statistical analysis confirmed the antigenotoxic and cytoprotective properties of Argovit™. These findings support its potential application as a mitigating agent in scenarios of environmental or occupational exposure to genotoxic compounds.

## 1. Introduction

The investigation of silver nanoparticles (AgNPs) has garnered significant attention in recent decades due to their antimicrobial and anticancer properties and potential applications in gene therapy [1,2]. AgNPs have shown unique properties that enable their interaction at the cellular and molecular levels, which has opened the possibility of using these nanomaterials to mitigate genotoxic damage caused by environmental and chemical toxicants [3,4,5,6]. Among these agents, sodium arsenite stands out as an environmental pollutant that has been widely studied for its adverse effects on human health, primarily due to its ability to induce genotoxic and cytotoxic damage in various cell types [7,8]. Specifically, Argovit™ AgNPs are a widely studied formulation due to their remarkable antimicrobial properties and potential applications in biomedical fields [9,10,11]. Developed by Russian researchers, Argovit™ nanoparticles are notable for their protective polyvinylpyrrolidone (PVP) coating, which improves their stability and reduces their toxicity compared to other silver nanoparticles [12]. This coating enables more precise control of their interactions with cells and tissues, making them particularly attractive for preclinical studies [12,13]. Although AgNPs have been shown to possess antioxidant properties and act as modulators of oxidative stress, their impact on cytotoxicity and genotoxicity has been the subject of debate [14]. Several studies have explored the effects of Argovit™ nanoparticles in cellular models, finding that, although at low concentrations, they can reduce genotoxic damage, at high concentrations, they can induce cytotoxic and genotoxic effects in different cell types [2,11,15]. However, the results vary according to the experimental conditions and cell types used, highlighting the importance of further research on their safety and mechanisms of action in specific contexts, such as interaction with genotoxic agents like sodium arsenite [10,16,17].

Arsenic is a naturally occurring metalloid found ubiquitously in the environment in various chemical forms, like sodium arsenite, one of its most toxic variants. According to the U.S. Agency for Toxic Substances and Disease Registry (ATSDR), arsenic ranks first on the list of the 10 most hazardous substances due to its known mutagenic, carcinogenic, and teratogenic effects [18]. These effects have been extensively documented in epidemiological and experimental studies, which have shown that prolonged exposure to arsenic, even at low levels, can lead to the development of several types of cancer, such as skin, lung, bladder, and kidney cancer [18,19].

Sodium arsenite is a soluble compound that can quickly enter the human body through ingestion of contaminated water, inhalation, or dermal contact, directly affecting cell function. One of the main characteristics of arsenic is its ability to induce DNA damage, including strand breaks, chromosomal aberrations, and the formation of micronuclei in exposed cells. These alterations are indicative of genotoxicity, a phenomenon that precedes mutagenesis and the development of malignant neoplasms [18,20].

Sodium arsenite acts as a cytotoxic and genotoxic agent, affecting various types of cells, including human lymphocytes, which play a crucial role in the immune response, playing a vital role in defending against foreign invaders and abnormal cells, including bacteria, viruses, and cancer cells [19,21]. At the cellular level, sodium arsenite generates reactive oxygen species (ROS), which trigger oxidative stress, alterations in cell signaling pathways, and DNA structural damage. This damage can manifest as micronuclei, DNA strand breaks, and other markers of genomic instability, which contribute to cell transformation and the development of cancer [22,23].

Several studies have confirmed that sodium arsenite is a potent mutagen and possesses teratogenic properties, affecting embryonic development in exposed organisms. The cytotoxic effects of arsenic include induction of apoptosis, necrosis, and mitochondrial dysfunction, which compromises cell viability. In experimental models, prolonged exposure to this compound has shown a clear correlation with decreased cell proliferation and increased markers of programmed cell death [19,24,25,26].

Silver nanoparticles (AgNPs) have shown antioxidant properties [27] and are protective against cellular damage, particularly when confronted with reactive oxygen species (ROS) induced by toxic agents [28,29]. Several studies have highlighted the ability of AgNPs to interfere with the ROS generation pathways, thereby reducing cellular oxidative stress and, consequently, DNA damage [15]. However, the interaction of AgNPs with toxic agents such as sodium arsenite has not been fully elucidated. Recent studies suggest that Argovit™ AgNPs may exert a protective role when applied after exposure to genotoxic compounds, offering a potential alternative for their use as therapeutic agents in the prevention of genetic damage.

The cytokinesis-blocking micronucleus assay (CBMN) has been widely used for measuring genotoxicity in human and animal cells [30]. This assay is particularly effective in identifying chromosomal or mitotic damage resulting in the formation of micronuclei in binucleated cells [31]. Traditionally, this assay is performed over 72 h; however, recent studies have explored modifications to the protocol [32], such as incubation time, which may allow for better observation of genotoxic effects at late cell stages or under specific experimental conditions [33]. In the context of the present study, modifying the protocol to 144 h offers the opportunity to evaluate the long-term effects of Argovit™ AgNPs after exposure to sodium arsenite and to investigate how these particles may contribute to reducing genotoxic damage.

Studies on the interaction between silver nanoparticles and arsenic have primarily focused on technological applications, such as the use of silver nanoparticles in the construction of nano filters for the detection and separation of arsenic in effluents and contaminated soils [34,35]. These investigations have shown promising results in the field of environmental remediation, where the adsorptive and antimicrobial properties of nanoparticles play a crucial role [36,37]. However, very few studies have investigated the interaction of silver nanoparticles and arsenic in biological models, either in vivo or in vitro. The paucity of research in this area leaves a significant gap in knowledge about the biological effects of this interaction, especially its cytotoxic and genotoxic potential in human cells. This lack of study highlights the need to investigate the health effects of silver nanoparticles further, particularly when combined with genotoxic compounds such as sodium arsenite.

The current literature is still limited in terms of studies evaluating the specific interaction between AgNPs and sodium arsenite in cellular models [38,39]. Some preliminary work has shown that AgNPs can partially neutralize damage induced by heavy metals and toxic compounds by reducing oxidative stress and enhancing DNA repair mechanisms [21,39].

Silver nanoparticles have appeared as promising candidates due to their unique physicochemical and biological properties in the search for potential agents capable of mitigating arsenite-induced genotoxicity [38]. Among these, Argovit™ AgNPs stand out for their well-documented safety profile and proven protective effects in various biological models. Earlier toxicological evaluations following OECD Guideline 420 have reported low acute oral toxicity for Argovit™ AgNPs, with LD50 values placing them in Category 4 of the Globally Harmonized System [40]. Studies in mice have shown no significant damage and non-genotoxic and even genoprotective effects in in vivo models exposed to cytosine arabinoside and cyclophosphamide [41,42]. Environmental studies have also demonstrated low cytotoxicity in plant systems [43]. Furthermore, Argovit™ has shown favorable biocompatibility in human erythrocytes and lymphocytes [10,41,42] and has been applied successfully in clinical contexts, including SARS-CoV-2 prevention, diabetic foot ulcer treatment, and respiratory care [44]. These characteristics make Argovit™ a particularly suitable candidate for evaluating its potential to mitigate arsenite-induced cytogenotoxicity in human lymphocytes [45].

In this regard, it is relevant to investigate whether the application of AgNPs after exposure to sodium arsenite can mitigate the cytotoxic and genotoxic effects, particularly in human lymphocytes. The present work aims to contribute to the understanding of the protective mechanisms of Argovit™ AgNPs when applied after exposure to a genotoxic agent such as sodium arsenite [35,36,37,38]. By using a modified design of the cytokinesis-blocking micronucleus assay, this study aims to expand knowledge on the potential of silver nanoparticles to mitigate cell damage and their potential use in preventive therapeutic strategies for environmental and occupational exposure to genotoxic compounds. This study hypothesizes that Argovit™ AgNPs reduce the cytotoxic and genotoxic effects induced by sodium arsenite in human lymphocytes when applied after exposure to the toxic agent. The objective is to evaluate the impact of Argovit™ silver nanoparticles on the reduction of sodium arsenite-induced cytotoxicity and genotoxicity in human lymphocytes using a modified cytokinesis-blocking micronucleus assay at 144 h.

## 2. Materials and Methods

### 2.1. Preparation and Characterization of Argovit™ Silver Nanoparticles

The silver nanoparticles used in this study correspond to the Argovit™ formulation donated by Dr. Vasily Burmistrov from the Vector-Vita Scientific and Production Center (Novosibirsk, Russia). This formulation consists of a stable aqueous suspension containing 1.2% elemental silver by weight, stabilized with 18.8% polyvinylpyrrolidone (PVP; 12.6 ± 2.7 kDa). Its physicochemical properties have been thoroughly characterized, including spheroidal morphology with an average diameter of 35 ± 12 nm (TEM), a hydrodynamic diameter of approximately 70 nm (DLS), a zeta potential of −15 mV, and a surface plasmon resonance peak at 420 nm [46]. These parameters were used as quality control indicators for batch consistency throughout the experimental period. No aggregation or instability was observed during storage or upon dilution in the culture medium. Colloidal stability, negative surface charge, and PVP coating are considered critical for the biocompatibility and biological performance of the nanoparticles, particularly regarding their interaction with cellular membranes and internalization dynamics in ex vivo systems [46].

### 2.2. Sodium Arsenite Treatment

Sodium arsenite (NaAsO_2_, Sigma-Aldrich, St. Louis, MO, USA) was prepared in an aqueous solution at a concentration of 10 mM and diluted in a cell culture medium to achieve a final concentration of 3.7 × 10^−3^ µg/mL in the treatments. Specifically, the sodium arsenite concentration of 3.7 × 10^3^ µg/mL was previously validated in our CBMN assays as a genotoxic positive control, consistently inducing chromosomal damage [10]. This dose is within the range reported in the literature for inducing micronuclei formation in human lymphocytes without causing extensive cytotoxicity or cell cycle arrest [10].

### 2.3. Human Lymphocyte Culture

The cells used in this culture were derived from three independent samples (0.5 mL each) of venous whole blood obtained from a single healthy adult donor (aged 25–35 years, non-smoker, with no known exposure to genotoxic agents, medications, or chronic diseases in the preceding three months) via venipuncture. All procedures were conducted according to the guidelines of the Bioethics Committee of the Faculty of Medicine and Psychology at the Autonomous University of Baja California, Tijuana (approval number 05/2014-1). Blood samples were anticoagulated with heparin and cultured directly as whole blood in RPMI-1640 medium supplemented with 15% fetal bovine serum, penicillin (100 U/mL), and streptomycin (100 µg/mL). Lymphocyte proliferation was stimulated by adding phytohemagglutinin (PHA) at a final concentration of 5 µg/mL, and cultures were incubated at 37 °C in a 5% CO_2_ atmosphere. The use of whole blood without prior lymphocyte isolation preserved the physiological milieu, allowing plasma components and other blood factors to modulate cellular responses. This ex vivo approach is consistent with established protocols for the cytokinesis-block micronucleus (CBMN) assay in human lymphocytes and enhances the findings’ biological relevance and translational value [47].

### 2.4. Modifications of the Culture Medium

**Figure 1:**
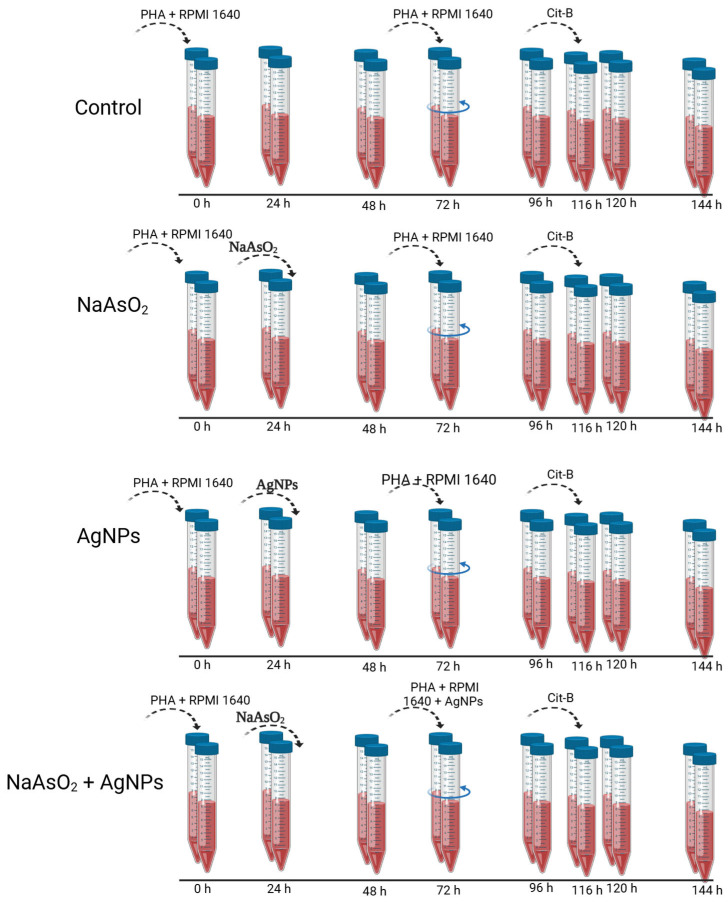
Schematic representation of the timeline and experimental conditions for each treatment group included in the CBMN assay. Each condition was carried out in duplicate, as indicated by the presence of two Falcon tubes per time point. In the Control group, lymphocyte cultures were stimulated with PHA and maintained in RPMI 1640 medium throughout the experiment. In the NaAsO_2_ group, sodium arsenite was added 24 h after stimulation. In the AgNPs group, silver nanoparticles were added at 24 h post-stimulation. In the NaAsO_2_ + AgNPs group, sodium arsenite was added at 24 h, followed by silver nanoparticles at 72 h. PHA: phytohemagglutinin; Cit-B: cytochalasin B.

After 72 h of culture, the cells were centrifuged, and 5 mL of medium was removed. Fresh RPMI-1640 medium supplemented with PHA at five µg/mL was added. Subsequently, the cells were incubated for an additional 72 h, bringing the total incubation time to 144 h from the start of the experiment (Figure 1).

### 2.5. Cytokinesis-Blocking Micronucleus Assay

The cytokinesis-blocking micronucleus assay was performed according to the standard procedure described by [47], with one significant modification: the total incubation time was extended to 144 h, rather than the original 72 h. The test substances (sodium arsenite and/or silver nanoparticles) were added 24 h after the start of the culture [48,49]. In the corresponding group, the second test substance (sodium arsenite or silver nanoparticles) was added 72 h after the start of the culture. At 116 h, cytochalasin B (Sigma-Aldrich, St. Louis, MO, USA) was added at a final concentration of 6 µg/mL to inhibit cytokinesis, allowing the formation of binucleated cells. After a total incubation period of 144 h, the cells were collected by centrifugation and fixed in a 3:1 methanol/acetic acid solution. The samples were stained with eosin and methylene blue and viewed under an optical microscope to count cytogenotoxicity biomarkers.

**Figure 2:**
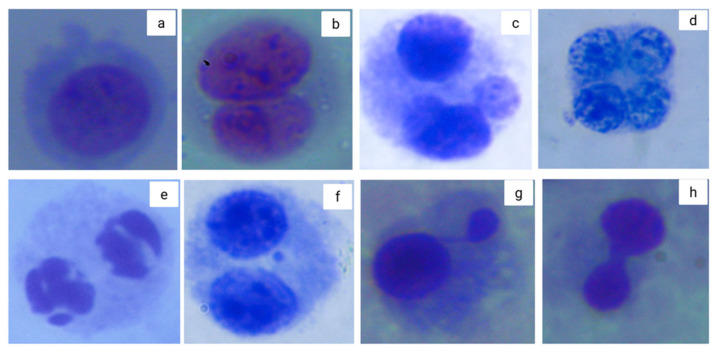
Representative nuclear morphologies and cytogenetic abnormalities found in cultured human lymphocytes using the cytokinesis-block micronucleus (CBMN) assay. Images were acquired with a Primo Star microscope (Carl Zeiss, Oberkochen, Germany) (Carl Zeiss, Oberkochen, Germany) using a 100× oil immersion objective and a Hayer digital camera. (**a**) Mononucleated cell; (**b**) binucleated cell; (**c**) trinucleated cell; (**d**) tetranucleated cell; (**e**) apoptotic cell exhibiting chromatin condensation and nuclear fragmentation; (**f**) binucleated cell with a micronucleus (MNi); (**g**) cell displaying a nuclear bud (NBud), indicative of gene amplification or DNA repair; (**h**) cell with a nucleoplasmic bridge (NPB), associated with misrepair of DNA strand breaks or dicentric chromosome formation. Micrographs by Balam Ruiz.

Representative nuclear morphologies and cytogenetic abnormalities seen in the CBMN assay are shown in Figure 2. These include mononucleated, binucleated, multinucleated, and apoptotic cells, as well as micronuclei (MNi), nuclear buds (NBuds), and nucleoplasmic bridges (NPBs) [50].

### 2.6. Experimental Design

The experimental design was based on a modified version of the cytokinesis-block micronucleus (CBMN) assay originally proposed by Fenech and Morley [47], extended to 144 h to allow analysis across two complete lymphocyte cell cycles and capture delayed cytogenotoxic effects. This extended protocol is supported by the findings of Whitwell et al. (2015), who demonstrated that incorporating a more extended incubation period and medium renewal improves the stability and sensitivity of the assay in peripheral blood lymphocytes [48].

As illustrated in Figure 1, four experimental groups were established using 0.5 mL of venous whole blood cultured ex vivo for 144 h [48]:

(1) Control group: Phytohemagglutinin (PHA) and RPMI-1640 medium were added at 0 h to stimulate lymphocyte proliferation. At 72 h, the medium was partially replaced with fresh RPMI + PHA. Cytochalasin B (6 µg/mL) was added at 116 h to block cytokinesis, and the cultures were harvested at 144 h. This group establishes the baseline cellular behavior without exposure to toxicants or nanoparticles.
(2) Sodium arsenite (NaAsO_2_) group: Identical to the control group, with the addition of sodium arsenite (3.7 × 10^−^^3^ µg/mL) at 24 h. This group serves as a positive control for genotoxicity based on the well-documented ability of NaAsO_2_ to induce chromosomal damage in human lymphocytes.
(3) AgNPs group: AgNPs (3.6 × 10^−^^3^ µg/mL) were added at 24 h under identical conditions to the control. This group was designed to assess the intrinsic cytogenotoxic potential of Argovit™ AgNPs at a sub-toxic concentration previously validated in our group’s earlier work [10].
(4) NaAsO_2_ + AgNPs group (post-treatment): PHA and RPMI-1640 were added at 0 h. Sodium arsenite was added alone at 24 h to induce cytogenotoxic damage. At 72 h, the medium was refreshed and supplemented with PHA and AgNPs (3.6 × 10^−^^3^ µg/mL). This group aimed to evaluate whether post-exposure application of AgNPs could mitigate the damage induced by NaAsO_2_, simulating a potential therapeutic or recovery model. Cytochalasin B was added at 116 h, and cells were harvested at 144 h.

In all groups, additional medium refreshment steps were performed at 96 h and 120 h to maintain cell viability and nutrient availability throughout the extended culture period.

All experimental conditions were performed in duplicate, following the recommendations described in the standardized CBMN assay protocol by Fenech [47,48,49,50,51] to ensure assay reproducibility and minimize intra-assay variability.

### 2.7. Statistical Analysis

All experimental conditions were performed in duplicate, following the recommendations established in the CBMN assay [51], which suggests duplicate cultures to enhance internal validity and reproducibility in cytogenetic evaluations using human lymphocytes.

Five hundred cells were scored for each replicate to determine the nuclear division index (NDI) and the percentage of apoptotic cells. In comparison, 1000 binucleated cells were scored to assess genotoxic biomarkers, specifically micronuclei (MNi), nucleoplasmic bridges (NPBs), and nuclear buds (NBuds).

The NDI was calculated using the following formula:

$$NDI=\frac{M_{1}+{2M}_{2}+{3M}_{3}+{4M}_{4}}{N}$$

where *M*_1_–*M*_4_ represent the number of cells with 1 to 4 nuclei, respectively, and *N* is the total number of viable cells scored.

Given the discrete and often Poisson-like distribution of cytogenetic endpoints, the data were initially assessed for variance homogeneity using Bartlett’s test. Due to observed heterogeneity, data were transformed to better approximate normality.

Intergroup comparisons were conducted using the non-parametric Kruskal–Wallis test and Dunn’s post hoc test for pairwise comparisons. A *p*-value of <0.05 was considered statistically significant.

All statistical analyses and graphical representations were performed using Jamovi software (version 2.5) and GraphPad Prism (version 9.0).

## 3. Results

This study evaluated the effect of Argovit™ silver nanoparticles on sodium arsenite-induced cytotoxicity and genotoxicity in human lymphocytes ex vivo. The results demonstrated a clear reduction in cytotoxic effects in lymphocytes treated with silver nanoparticles following sodium arsenite exposure, compared to untreated controls. Analysis using the L-CBMN assay revealed a significant decrease in micronucleus formation in binucleated cells treated with silver nanoparticles. Detailed results for each experimental group, along with the statistically significant differences between treatments, are provided in Supplementary Table S1 and further illustrated in the figures described in the following sections.

### 3.1. Cytotoxicity

**Figure 3:**
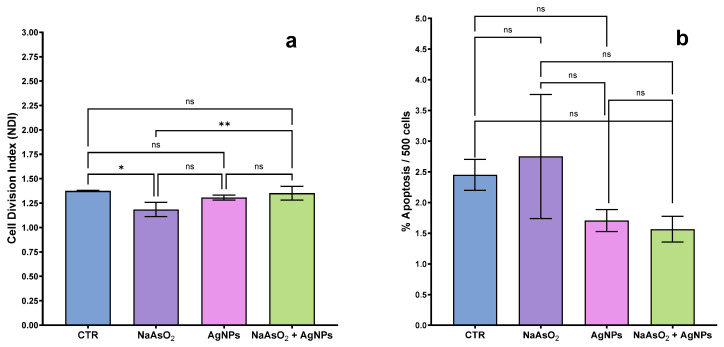
Cytotoxicity biomarkers in human lymphocytes exposed to sodium arsenite (NaAsO_2_), silver nanoparticles (AgNPs), and their combination: (**a**) Cell division index (NDI); (**b**) apoptosis frequency. * *p* < 0.05; ** *p* < 0.01; ns = not significant.

In the cell division index (Figure 3a), significant differences are observed between the control group (CTR) and the group that received sodium arsenite (As), indicating that sodium arsenite has a substantial effect on cell division compared to the control. The other treatments, including silver nanoparticles (AgNPs) and the combination of sodium arsenite with silver nanoparticles (As + AgNPs), show no significant difference from the control, suggesting that nanoparticles may have a protective effect or, at the very least, do not exacerbate the cytotoxic effect of sodium arsenite. Dunn’s multiple testing shows a significant difference between the CTR and the As group (*p* < 0.05, **).

The fact that there is no significant apoptosis (Figure 3b) suggests that the mechanism of damage caused by sodium arsenite is more focused on interfering with the cell’s ability to divide, possibly through genotoxic damage that arrests the cell cycle, rather than directly activating apoptotic pathways. By not increasing apoptosis, silver nanoparticles could be acting to counteract damage without triggering programmed cell death (Figure 3b).

This could be relevant if you are evaluating the safety or protective effects of silver nanoparticles, as their effect does not appear to include the activation of apoptosis, which could be favorable in a context where you aim to reduce cytotoxicity without promoting cell death.

### 3.2. Genotoxicity

Regarding micronuclei (MNi), nucleoplasmic bridges (NPBs), and nuclear buds (NBuds), the former can be said to represent clastogenic or aneuploidogenic events [51]. NPBs indicate defects in the organization of the mitotic spindle, while NBuds have been related to the cell’s attempt to repair the DNA damage [51,52].

**Figure 4:**
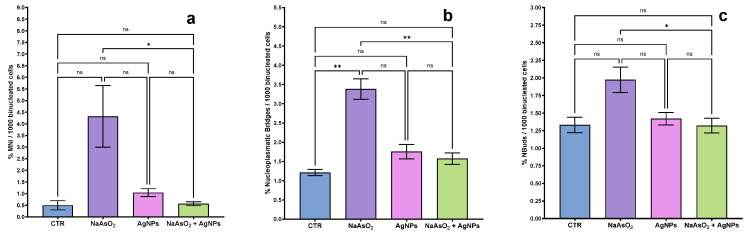
Frequency of genotoxic biomarkers: (**a**) micronuclei (MNi), (**b**) nucleoplasmic bridges (NPBs), and (**c**) nuclear buds (NBuds) in human lymphocytes. Sodium arsenite significantly increased all biomarkers. Post-treatment with AgNPs at 72 h reduced their frequency (*p* < 0.05). * indicates statistically significant differences (*p* < 0.05); ** indicates highly significant differences (*p* < 0.01); ns = not significant.

Figure 4, which shows the percentage of the three biomarkers of genotoxicity when enlarged, illustrates pixelation. It is observed that sodium arsenite treatments affect the genetic material of cells, causing clastogenesis or aneuploidy (Figure 4a) due to the high percentage of MNi. On the other hand, sodium arsenite leads to defects in chromosome segregation, resulting in the formation of NPBs (Figure 4b) and NBuds (Figure 4c), as well as AgNPs, although without significant differences (*p* > 0.05). In contrast, the addition of AgNPs at 72 h led to a significant decrease (*p* < 0.05) in the frequency of micronuclei, NPBs, and NBuds, suggesting that this treatment mitigates sodium arsenite-induced genotoxic damage.

## 4. Discussion

This study highlights the potential protective role of Argovit™ silver nanoparticles against cytotoxic and genotoxic damage induced by sodium arsenite in cultured human lymphocytes, mainly when administered after exposure to the toxicant. While no significant differences in apoptosis were observed among the experimental groups, the findings suggest that sodium arsenite predominantly affects cell proliferation by disrupting the cell cycle rather than directly triggering programmed cell death. The observed reduction in nuclear division index (NDI) indicates a loss of proliferative capacity, consistent with a cytostatic rather than cytotoxic mechanism [53].

The observed decrease in NDI supports the notion that sodium arsenite impairs cell cycle progression, possibly by arresting the cell cycle at the G1 or G2/M checkpoints [8,54]. Such an arrest may delay or obscure the appearance of key biomarkers, such as micronuclei (MNi), nucleoplasmic bridges (NPBs), or nuclear buds (NBuds), which are typically detected in proliferating cells. This phenomenon has been previously described and is consistent with the idea that cell cycle inhibition can lead to an underestimation of DNA damage and apoptosis in genotoxicity assays [51,55,56].

The findings align with previous studies indicating that cell cycle arrest can interfere with detecting genotoxic damage, both by delaying the formation of visible damage indicators and by reducing the number of cells available for analysis. Moreover, the combination of sodium arsenite with silver nanoparticles appears to mitigate cytotoxic effects but may not eliminate genotoxic risks [41,42]. This dual behavior suggests a nuanced interaction between both agents, where nanoparticles may support repair mechanisms without entirely preventing damage [57].

The detection of NPBs in the arsenite-only group indicates interference with mitotic spindle assembly and chromosome segregation. In contrast, the lack of significant NPB formation in the AgNPs-treated groups suggests a protective role or neutral behavior of the nanoparticles in this context. Similarly, the presence of NBuds in multiple groups suggests an active DNA repair process, which the presence of AgNPs may enhance, although it does not necessarily lead to complete repair [57,58].

A significant increase in the nuclear division index (NDI) was observed in the group treated with Argovit™ silver nanoparticles after sodium arsenite exposure, compared to the group exposed to sodium arsenite alone (*p* < 0.05), as shown in Figure 2a. This recovery in NDI suggests a partial restoration of proliferative capacity and indicates that the cytostatic effect of arsenite was mitigated. In parallel, a significant reduction in the frequency of genotoxicity biomarkers—micronuclei (MNi), nuclear buds (NBuds), and nucleoplasmic bridges (NPBs)—was detected in the same group (*p* < 0.05 for all), in contrast to the elevated frequencies induced by arsenite alone. These findings support the hypothesis that Argovit™ AgNPs not only reduce cytotoxic damage but also diminish chromosomal instability and nuclear anomalies associated with genotoxic stress [42].

The protective effect observed may be explained, at least in part, by the capacity of AgNPs to modulate oxidative stress pathways. Sodium arsenite is known to induce DNA damage by generating reactive oxygen species (ROS), disrupting mitotic spindle formation and promoting chromosomal aberrations. At subcytotoxic concentrations, Argovit™ AgNPs may enhance the activity of cellular antioxidant systems, particularly those dependent on glutathione (GSH). Prior studies by our group have demonstrated that Argovit™ AgNPs can stimulate GSH-mediated responses, potentially increasing the activity of glutathione peroxidase and glutathione reductase, which are critical for neutralizing ROS and preserving redox homeostasis [42].

In addition, the surface properties of Argovit™ AgNPs, coated with polyvinylpyrrolidone (PVP), favor the formation of a biocorona when exposed to serum proteins, which modulates nanoparticle–cell interactions [59,60,61,62]. This protein corona may reduce nonspecific binding, cellular uptake, and intracellular stress, contributing further to the observed decrease in cytogenotoxic biomarkers [60,62]. The reduction in NBuds and NPBs may also reflect improved DNA repair and proper chromosome segregation. Together, these results suggest that the post-treatment application of Argovit™ AgNPs counteracts arsenite-induced cytogenotoxicity by reinforcing antioxidant defenses, stabilizing nanoparticle behavior through biocorona formation, and enhancing genome integrity [61].

As this study employed an extended 144 h culture protocol, particular care was taken to reduce variability associated with long-term incubation, including the risks of culture fatigue, replication stress, or spontaneous DNA damage. To mitigate these effects, partial medium replacements were carried out at 72, 96, and 120 h, ensuring nutrient availability and reducing metabolic by product accumulation [59]. Additionally, untreated control cultures were kept under identical conditions as treated groups and consistently showed low frequencies of micronuclei, apoptosis, and other nuclear anomalies, suggesting minimal baseline damage. Blinded and randomized scoring further supported the reliability of the observed cytogenetic effects. While no specific molecular markers of proliferation, senescence, or early DNA damage were applied, the experimental design and internal consistency of the controls reinforce the interpretability of the findings under long-duration assay conditions [59].

Another consideration involves the potential physicochemical interaction between silver nanoparticles (AgNPs) and sodium arsenite (NaAsO_2_). Although in our experimental design, these compounds were administered sequentially—NaAsO_2_ at 24 h and AgNPs at 72 h—it is plausible that residual arsenite remained bioavailable in the culture medium and could have interacted with AgNP surfaces or with released Ag_+_ ions. Such interactions may lead to the formation of Ag–As complexes, adsorption phenomena, or changes in ionic speciation, all of which could influence bioavailability and toxicodynamics. However, the PVP coating of Argovit™ AgNPs likely modulates their surface reactivity and colloidal behavior, possibly reducing direct reactivity with metalloid species [62,63]. Nonetheless, under conditions such as low pH or oxidative stress, AgNPs have been shown to adsorb, transform, or reduce metal(loid) ions, including arsenic, in environmental and biological contexts. These potential interactions were not assessed in the present study. Still, they should be evaluated in future research using UV–Vis spectroscopy, zeta potential analysis, inductively coupled plasma mass spectrometry (ICP-MS), or X-ray photoelectron spectroscopy (XPS). Such methods would offer valuable insight into speciation dynamics and help determine whether the observed protective effects of AgNPs are mediated by biological modulation, physicochemical sequestration, or both.

Extending the culture time from 72 to 144 h allowed for the analysis of two cell cycles but may have introduced additional variables. Prolonged exposure to nutrients like PHA and toxicants, even with the renewal of the culture medium, may cause cumulative cytotoxic and genotoxic effects, increased cell cycle arrest, and possibly cellular senescence [64]. These effects could lead to decreased NDI and influence the interpretation of proliferation and damage markers. In contrast, prolonged culture may offer more significant opportunities for DNA repair, though this would depend on the efficiency of the repair mechanisms and the severity of the damage [64]. These observations are supported by previous in vivo studies conducted by our group, in which the genoprotective potential of Argovit™ AgNPs was assessed in BALB/c mice exposed to well-known chemotherapeutic genotoxins. In other study by our group [41], silver nanoparticles mitigated cyclophosphamide-induced genotoxicity in peripheral blood erythrocytes, as showed by a reduction in micronucleated erythrocytes across multiple time points following repeated exposure. Similarly, in a more recent study, [42] proved that Argovit™ AgNPs exerted a protective effect against the genotoxicity induced by cytosine arabinoside (Ara-C), again using a time-course design that enabled monitoring of damage progression and repair [41,42].

These findings corroborate the time-dependent nature of AgNP action and strengthen the interpretation that the post-treatment administration of nanoparticles—such as in the current ex vivo model—may support delayed recovery processes. Together, these studies provide a robust experimental framework for incorporating kinetic and phase-specific evaluations in future in vitro assays using human lymphocytes. It is important to note that the CBMN assay is a terminal-point assay designed to capture chromosomal and nuclear anomalies after a defined culture period. Still, it does not allow real-time assessment of cell cycle dynamics or checkpoint activation. In our study, the observed reduction in the nuclear division index (NDI), along with the absence of significant apoptosis, is consistent with a cytostatic response, potentially due to interference with cell cycle progression. However, specific phase arrest (e.g., G1 or G2/M) cannot be confirmed without direct molecular or cytometric evidence.

Our laboratory does not currently have access to flow cytometry or cell sorting instrumentation, which constrains the inclusion of real-time cell cycle markers. Nonetheless, the decision to use the extended CBMN protocol (144 h) was strategic, as it allowed us to observe the accumulated outcome of proliferation disruption and chromosomal instability in a biologically relevant timeframe.

Future work may benefit from collaborative efforts or the incorporation of complementary molecular tools, such as immunostaining or RT-qPCR for key cell cycle regulators (e.g., cyclins, p21, Chk1/2), to confirm checkpoint engagement and further define the mechanisms underlying the protective effects of Argovit™ AgNPs. Meanwhile, the patterns observed in NDI, NBuds, and NPBs offer valuable insights into the delayed cytogenotoxic consequences of sodium arsenite exposure and the mitigating potential of nanoparticle intervention [41,42].

A significant strength of this study is the adaptation of the CBMN assay to a prolonged culture model, which enables the observation of effects across two complete cell cycles [65]. This provides a more comprehensive understanding of delayed cytogenetic effects. However, the more prolonged incubation also introduces challenges, such as culture fatigue, nutrient depletion, and increased variability, which could affect cell viability and mask treatment-related effects. While silver nanoparticles showed protective effects in reducing cytotoxicity, their role in combination with sodium arsenite appears complex and may even exacerbate genotoxic damage under certain conditions. This suggests the need for a cautious interpretation of nanoparticle safety, especially in co-exposure scenarios.

Despite these limitations, it is important to note that Argovit™ AgNPs have been extensively evaluated in both experimental and clinical contexts, demonstrating a favorable safety profile across multiple biological models and applications. Supporting evidence includes prior toxicological studies conducted under OECD Guideline 420, which reported oral LD50 values ranging from 1067 to 1806 mg/kg for metallic silver content, classifying these formulations in Category 4 of the Globally Harmonized System [40]. When considering the full nanoparticle formulation, including polyvinylpyrrolidone (PVP) as a stabilizer, the LD50 exceeds 17,000 mg/kg, placing Argovit™ in the “practically non-toxic” category.

Histopathological evaluations in mice have shown no significant tissue damage, with AgNP accumulation limited to the gastrointestinal tract [40]. Additionally, Argovit™ has demonstrated non-genotoxic behavior in melanoma models [65] and genoprotective effects against cytosine arabinoside and cyclophosphamide-induced damage in vivo [41,42]. Environmental studies have reported low cytotoxicity and genotoxicity in plant bioassays such as Allium cepa, supporting its safe application at low concentrations in agricultural contexts [15,66,67].

Argovit™ AgNPs have also shown low hemolytic activity in human erythrocytes from both healthy and diabetic donors, as well as low genotoxicity in human lymphocytes [10,68]. Clinical applications, including SARS-CoV-2 prevention [69] and diabetic foot ulcer treatment, further reinforce its biocompatibility [70,71]. Together, these findings highlight the high safety margin of Argovit™ AgNPs for both biomedical and environmental applications supporting their continued evaluation as potential mitigators of genotoxic damage.

## 5. Conclusions

This study suggests that Argovit™ silver nanoparticles may exert a partial protective effect against cytotoxic and genotoxic damage induced by sodium arsenite in cultured human lymphocytes. Under specific post-exposure treatment conditions, a significant recovery of the nuclear division index (NDI) and a reduction in the frequency of genotoxic biomarkers—including micronuclei (MNi), nucleoplasmic bridges (NPBs), and nuclear buds (NBuds)—were observed. These findings indicate a possible mitigation of chromosomal damage and a relative improvement in cellular proliferative dynamics, although further evidence is needed to establish the magnitude and specificity of the protective effect.

Adapting the cytokinesis-block micronucleus (CBMN) assay to a prolonged 144 h protocol allowed for the assessment of cumulative and delayed genotoxic responses and the potential reparative action of AgNPs. This methodological extension contributes meaningfully to investigating interactions between nanomaterials and genotoxic agents by expanding the analytical window to two complete cell cycles.

The observed effects may be mediated by antioxidant mechanisms involving glutathione, attenuation of oxidative stress, modulation of DNA damage response pathways, and formation of a biocorona that optimizes nanoparticle bioavailability while minimizing nonspecific reactivity. However, the absence of direct molecular markers of apoptosis, cell cycle arrest, or DNA repair limits the conclusiveness of these mechanistic interpretations.

Future studies should incorporate kinetic analyses at multiple time points, apoptosis and oxidative stress markers, and physicochemical characterization of potential interactions between arsenite and AgNPs. Additionally, long-term co-exposure models will be essential to assess genotoxic risk under chronic environmental or occupational scenarios involving nanomaterials and chemical contaminants.

## Data Availability

Data are available upon request from the corresponding author.

## Supplementary Materials

The following supporting information can be downloaded at: https://www.mdpi.com/article/10.3390/toxics13070539/s1, Table S1: Descriptive statistics for cytogenotoxicity biomarkers in human lymphocytes cultured ex vivo.

## Abbreviations

The following abbreviations are used in this manuscript:

LD	Linear dichroism
MNi	Micronucleus
NBuds	Nucleal buds
NPB	Nucleoplasmatic bridges
NDI	Nuclear Division Index
