# Supplementary material for: Argovit™ Silver Nanoparticles Mitigate Sodium Arsenite-Induced Cytogenotoxicity Effects in Cultured Human Lymphocytes
Source: Toxics. 2025 Jun 27;13(7):539. doi: 10.3390/toxics13070539 (PMC12297918; doi:10.3390/toxics13070539)
Supplement: Supplementary file 1 [file toxics-13-00539-s001.zip › toxics-3607608-supplementary.pdf]

Table S1. Descriptive Statistics for Cytogenotoxicity Biomarkers in Human Lymphocytes Cultured *Ex Vivo*

	n	Minimum	Maximum	Mean	Std. Deviation	Lower 95 % CI	Upper 95 % CI
**Nuclear Division Index**							
**CTR**	2	1.37	1.38	1.38	0.01	1.31	1.44
**$NaAsO_2$**	4	1.10	1.26	1.19	0.07	1.07	1.30
**AgNPs**	14	1.27	1.34	1.31	0.02	1.29	1.32
**$NaAsO_2$ + AgNPs**	10	1.20	1.45	1.35	0.07	1.30	1.40
**Apoptosis**							
**CTR**	2	2.20	2.70	2.45	0.35	-0.73	5.63
**$NaAsO_2$**	3	1.00	4.50	2.75	1.75	-1.60	7.10
**AgNPs**	12	1.00	2.80	1.71	0.62	1.32	2.10
**$NaAsO_2$ + AgNPs**	6	1.20	2.50	1.57	0.52	1.03	2.11
**Micronucleus**							
**CTR**	2	0.30	0.70	0.50	0.28	-2.04	3.04
**$NaAsO_2$**	2	3.00	5.65	4.33	1.87	-12.51	21.16
**AgNPs**	14	0.40	2.80	1.04	0.64	0.68	1.41
**$NaAsO_2$ + AgNPs**	9	0.20	1.00	0.57	0.24	0.38	0.76
**NBUDs**							
**CTR**	2	1.22	1.44	1.33	0.16	-0.07	2.73
**$NaAsO_2$**	4	1.54	2.27	1.97	0.36	1.40	2.55
**AgNPs**	14	0.98	2.19	1.42	0.33	1.23	1.61
**$NaAsO_2$ + AgNPs**	10	0.99	2.13	1.32	0.33	1.09	1.56
**Nucleoplasmatic Bridges**							
**CTR**	2	1.13	1.29	1.21	0.11	0.19	2.23
**$NaAsO_2$**	4	2.94	4.13	3.38	0.53	2.54	4.23
**AgNPs**	14	1.44	2.07	1.75	0.19	1.65	1.86
**$NaAsO_2$ + AgNPs**	10	0.88	2.44	1.57	0.48	1.23	1.92

This table summarizes the descriptive statistics (n, minimum, maximum, mean, standard deviation, and 95% confidence intervals) for each experimental group evaluated in this study. The biomarkers include the nuclear division index (NDI), percentage of apoptotic cells, micronuclei (MNi), nuclear buds (NBUDs), and nucleoplasmic bridges (NPBs), all assessed using the cytokinesis-block micronucleus (CBMN) assay after a 144-hour incubation period.
